# O-linked N-acetylglucosamine glycosylation of p65 aggravated the inflammation in both fibroblast-like synoviocytes stimulated by tumor necrosis factor-α and mice with collagen induced arthritis

**DOI:** 10.1186/s13075-015-0762-7

**Published:** 2015-09-14

**Authors:** Han Byeol Kim, Sang Won Lee, Chin Hee Mun, Ji Young Yoon, Jaeyoung Pai, Injae Shin, Yong Beom Park, Soo Kon Lee, Jin Won Cho

**Affiliations:** Department of Integrated OMICS for Biomedical Science, Graduate School, Yonsei University, 50 Yonsei-ro, Seodaemun–gu, Seoul 120-752 South Korea; Division of Rheumatology, Department of Internal Medicine, Yonsei University College of Medicine, 50 Yonsei-ro, Seodaemun–gu, Seoul 120-752 South Korea; Department of Chemistry, Yonsei University, 50 Yonsei-ro, Seodaemun–gu, Seoul 120-752 South Korea

## Abstract

**Introduction:**

We investigated the inflammatory potential of O-linked N-acetylglucosamine glycosylation (O-GlcNAcylation) of p65 in rheumatoid arthritis (RA).

**Methods:**

Fibroblast-like synoviocytes (FLS) and MH7A cells were treated with synthetic ThiaMet-G (200 μM), an O-GlcNAcase (OGA) inhibitor, followed by tumor necrosis factor (TNF)-α (10 μg/mL). Proliferation of synovial cells was measured by MTT assay, and the levels of mRNAs encoding pro-inflammatory molecules were quantitated by RT-PCR. The nuclear localization of O-GlcNAcylated of p65 and its DNA binding affinity and transcriptional activity were assessed. The severity assessment of arthritis and a histopathological examination were done in mice with collagen induced arthritis (CIA). ThiaMet-G (20 mg/kg) intraperitoneal injection was done every other day for 26 days. Fluorescence-activated cell sorting (FACS) analysis of T cells was performed.

**Results:**

Hyper-O-GlcNAcylation increased the proliferation and mRNA expression of pro-inflammatory genes in synoviocytes stimulated by TNF-α. Moreover, O-GlcNAcylation of p65 enhanced its proportion of nuclear localization, DNA binding affinity and transcriptional activity. In CIA mice, ThiaMet-G significantly aggravated the severity of arthritis clinically and histologically, and it also increased CD4 + IFN-γ + T cells and CD4 + IL-17+ T cells.

**Conclusions:**

O-GlcNAcylation of p65 increased the effects of TNF-α-mediated inflammation both *in vitro* (in synovial cells) and *in vivo* (in mice with CIA).

**Electronic supplementary material:**

The online version of this article (doi:10.1186/s13075-015-0762-7) contains supplementary material, which is available to authorized users.

## Introduction

O-linked N-acetylglucosamine (O-GlcNAc) glycosylation (O-GlcNAcylation) is a distinct form of glycosylation, which occurs in both the nucleus and the cytoplasm, unlike other forms of glycosylation, which are exclusive to either the endoplasmic reticulum or the Golgi complex [1]. O-GlcNAcylation of serine or threonine residues is mediated by O-GlcNAc transferase (OGT) and it is removed by O-GlcNAcase (OGA) under tightly regulated conditions [1]. O-GlcNAcylation affects numerous cellular processes, including embryonic development, basal metabolism, and tuning of the circadian clock, as well as immune responses in both cancer and inflammatory diseases [2–5]. Recently, O-GlcNAcylation of nuclear factor-kappa B (NF-κB) has attracted considerable attention, because of the reciprocal effects evident in different cell types, including positive regulation of pancreatic cancer cells and negative regulation of aortic smooth muscle cells [6–8].

Rheumatoid arthritis (RA) is an autoimmune disease characterized by inflammation and destruction of joint tissues [9]. As NF-κB acts as a pivotal regulator of pro-inflammatory molecules, such as tumor necrosis factor (TNF)-α and interleukin (IL)-6, in the pathogenesis of RA [9, 10], we hypothesized that O-GlcNAcylation of NF-κB might affect the course of RA. Here, we examined the inflammatory potential of O-GlcNAcylation of p65, a subunit of NF-κB, in RA. In vitro analyses were performed on fibroblast-like synoviocytes (FLS) and MH7A cells, followed by in vivo analyses using a mouse model of collagen-induced arthritis (CIA).

## Materials and methods

### Synoviocyte preparation and treatment

This study was approved by the Institutional Review Board of Severance Hospital (4-2012-0733). Informed consent was obtained from RA patients providing synovial tissues. FLS and MH7A cells were cultured as described previously [11, 12]. FLS and MH7A cells were treated with synthetic ThiaMet-G (200 μM), an OGA inhibitor, for 6 h, followed by TNF-α (10 μg/mL) for 12 h [13]. We synthesized ThiaMet-G according to a method for its synthesis provided by Prof. Vodadlo DJ (Simon Fraser University, Canada). The proliferation/viability of FLS and MH7A was measured by MTT assay.

### Overexpression and small hairpin RNA (shRNA)-mediated knockdown of OGA

The human OGA gene was cloned into a pRK5-FLAG vector (Genentech Inc., USA) and transiently transfected into MH7A cells using Lipofectamine 2000 (Invitrogen, USA). The pLKO.1 vector and packaging plasmid pMD.2G-VSVG and PsPax2 were used to generate a lentivirus-based OGA shRNA construct, as described previously [14]. This protocol is available on the website [15].

### RT-PCR

The levels of mRNAs encoding pro-inflammatory molecules were quantitated by RT-PCR. All experiments were performed in triplicate using a 7300 RT-PCR System (Applied Biosystems, USA) and a SYBR Green kit (Takara, Japan). A full list of primers is available in Additional file 1.

### Western blotting and succinylated wheat germ agglutinin (sWGA)-affinity purification

Western blotting immunoprecipitation was performed using anti-p65 (Santa Cruz Biotechnology, USA) and anti-O-GlcNAc (Covance, USA) and succinylated wheat germ agglutinin (sWGA)-affinity purification were performed for precipitating the *O-*GlcNAcylated proteins [16].

### Measurement of DNA binding affinity and transcriptional activity of p65

Avidin-biotin complex DNA (ABCD) lysis buffer containing 10 mM Tric-Cl (pH 7.4), 150 mM NaCl, 1 mM EDTA and 0.2 % Nonidet P-40 with protease inhibitor cocktail was used for MH7A cells lysis to measure DNA binding affinity. Lysates were incubated with biotinylated NF-κB promoter site DNA and streptavidin beads, washed three times in ABCD lysis buffer, and eluted in SDS sample buffer. NF-κB transcriptional activity was measured in MH7A cells transfected with NF-κB-dependent luciferase reporter gene construct 4xkB- Luc. Reporter gene assays were performed using the Dual-Luciferase kit (Promega, USA), with firefly luciferase activity normalized to renillia luciferase activity.

### Mice with CIA

We evenly divided 30 male DBA/1 mice at 8 weeks of age (SLC, Shizoka, Japan) into three groups (group 1, controls; group 2, untreated; group 3, ThiaMet-G-treated). We established a CIA model and assessed its severity clinically (arthritis score and paw thickness) and histologically (synovial proliferation, bone destruction and inflammatory cell infiltration under hematoxylin and eosin stain), as described previously [17]. Two weeks after primary immunization, mice were treated with 20 mg/kg ThiaMet-G via intraperitoneal (IP) injection, every other day for 26 days [18]. Controls and untreated mice were injected IP with the same volume of PBS every other day during the ThiaMet-G treatment period. Mice were next sacrificed and immune cells isolated from the spleen; T cells were then isolated and analyzed using fluorescence-activated cell sorting (FACS) [19]. The experimental protocols were approved by the Institutional Animal Care and Use Committee of Yonsei University College of Medicine.

### Statistical analyses

Each experiment was performed in triplicate, and repeated three to four times. All of the analyses were conducted using the SPSS package for Windows version 20.0 (SPSS Inc., Chicago, IL, USA). Data are representative of the mean values obtained. Differences between groups were evaluated using the Mann–Whitney *U* test. Multiple-group comparisons were performed using analysis of variance followed by application of Tukey’s method for multiple comparisons. *P* values <0.05 were considered to indicate statistical significance for all statistical evaluations.

## Results

### The effect of Hyper-O-GlcNAcylation on FLS growth

**Fig. 1:**
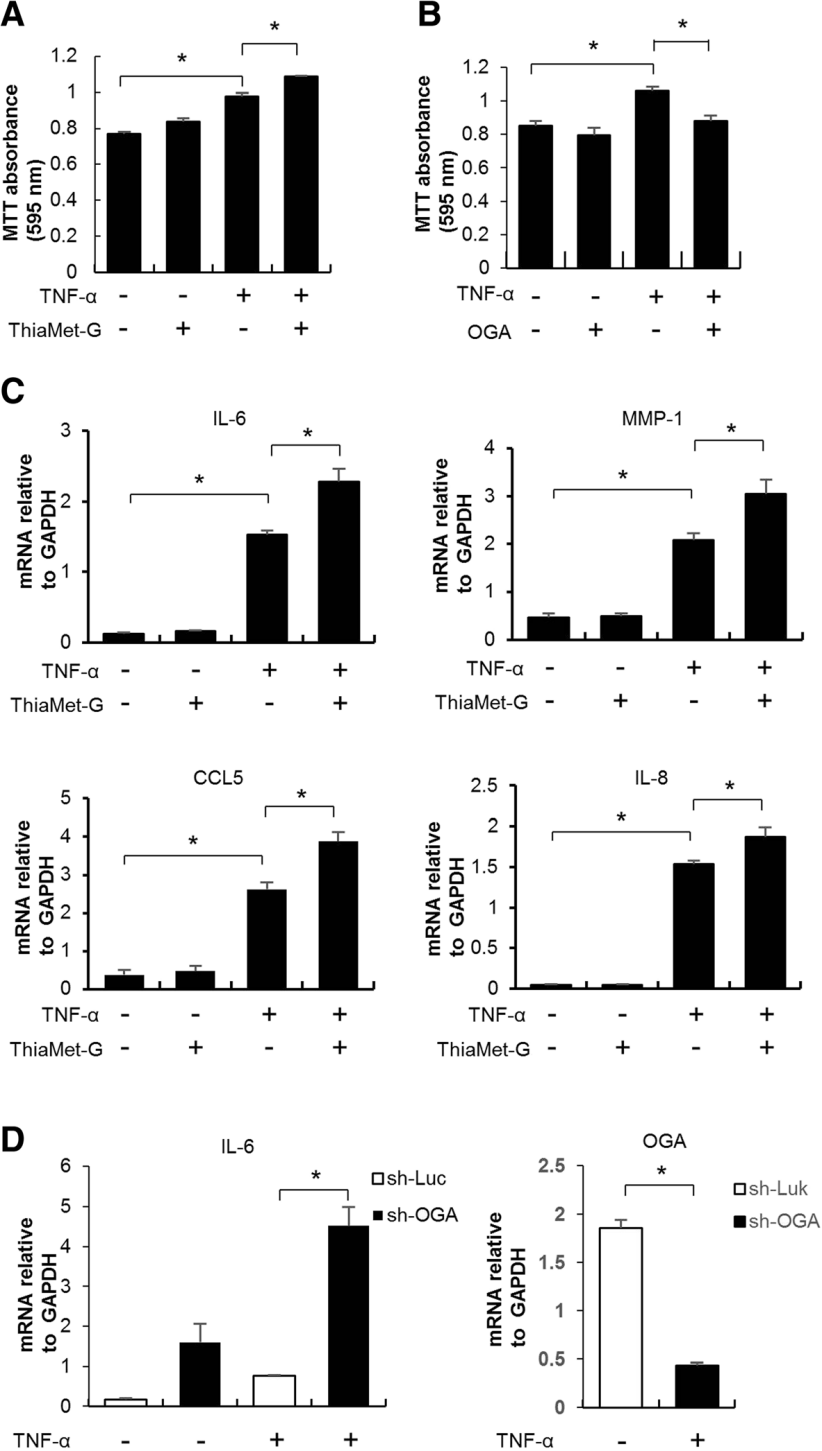
Effects of O-linked N-acetylglucosamine glycosylation on proliferation and gene expression in synovial cells. Fibroblast-like synoviocytes (FLS) and MH7A cells were treated with synthetic ThiaMet-G (200 μM) for 6 h, an O-GlcNAcase (OGA) inhibitor, followed by TNF-α (10 μg/mL) for 12 h. Proliferation of synovial cells was measured by MTT assay. **a** TNF-α significantly increased proliferation of FLS, compared to controls and proliferation was further enhanced following treatment with ThiaMet-G. **b** The human OGA gene was cloned into a pRK5-FLAG vector and transiently transfected into MH7A cells. In contrast to treatment of ThiaMet-G, overexpression of OGA in FLS significantly attenuated the effects of TNF-α on proliferation, compared to controls. **c** The levels of mRNAs encoding pro-inflammatory molecules including matrix metalloproteinase-1 (*MMP-1*), chemokine ligand 5 (*CCL5*) and IL-8, were quantitated by RT-PCR. ThiaMet-G did not affect the expression levels of pro-inflammatory molecules. But, ThiaMet-G significantly increased expression levels in TNF-α-stimulated FLS, compared to untreated controls. **d** The pLKO.1 vector and packaging plasmid pMD.2G-VSVG were used to generate a lentivirus-based OGA shRNA construct. OGA shRNA significantly decreased the expression of OGA by about 75 %, compared to sh-luciferase (*sh-Luc*) control. Similar effects were seen following shRNA-mediated knockdown of OGA, resulting in substantial increase in IL-6 expression, compared to those of sh-Luc controls in TNF-α-stimulated MH7A cells (*P <0.05). *GAPDH* glyceraldehyde-3-phosphate dehydrogenase

TNF-α significantly increased proliferation of FLS, compared to controls, and proliferation was further enhanced following treatment with ThiaMet-G (Fig. 1a). Similar effects were also evident following treatment with NButGT, another OGA inhibitor, in TNF-α-stimulated FLS (Additional file 2). In contrast, overexpression of OGA in FLS significantly attenuated the effects of TNF-α on proliferation, compared to controls (Fig. 1b).

### The effect of hyper-O-GlcNAcylation on pro-inflammatory gene expression

ThiaMet-G did not affect the expression levels of pro-inflammatory molecules, including IL-6, matrix metalloproteinase-1 (MMP-1), chemokine ligand 5 (CCL5) and IL-8 in unstimulated FLS [11]. However, ThiaMet-G significantly increased expression levels in TNF-α-stimulated FLS, compared to untreated controls (Fig. 1c). OGA shRNA significantly decreased the expression of OGA by about 75 %, compared to sh-Luc control. And similar effects were seen following shRNA-mediated knockdown of OGA, resulting in substantial increase in IL-6 expression, compared to those of sh-luciferase (sh-Luc) controls in TNF-α-stimulated MH7A cells (Fig. 1d).

### The effect of O-GlcNAcylation of p65 on nuclear localization, DNA binding affinity and transcriptional activity of p65

**Fig. 2:**
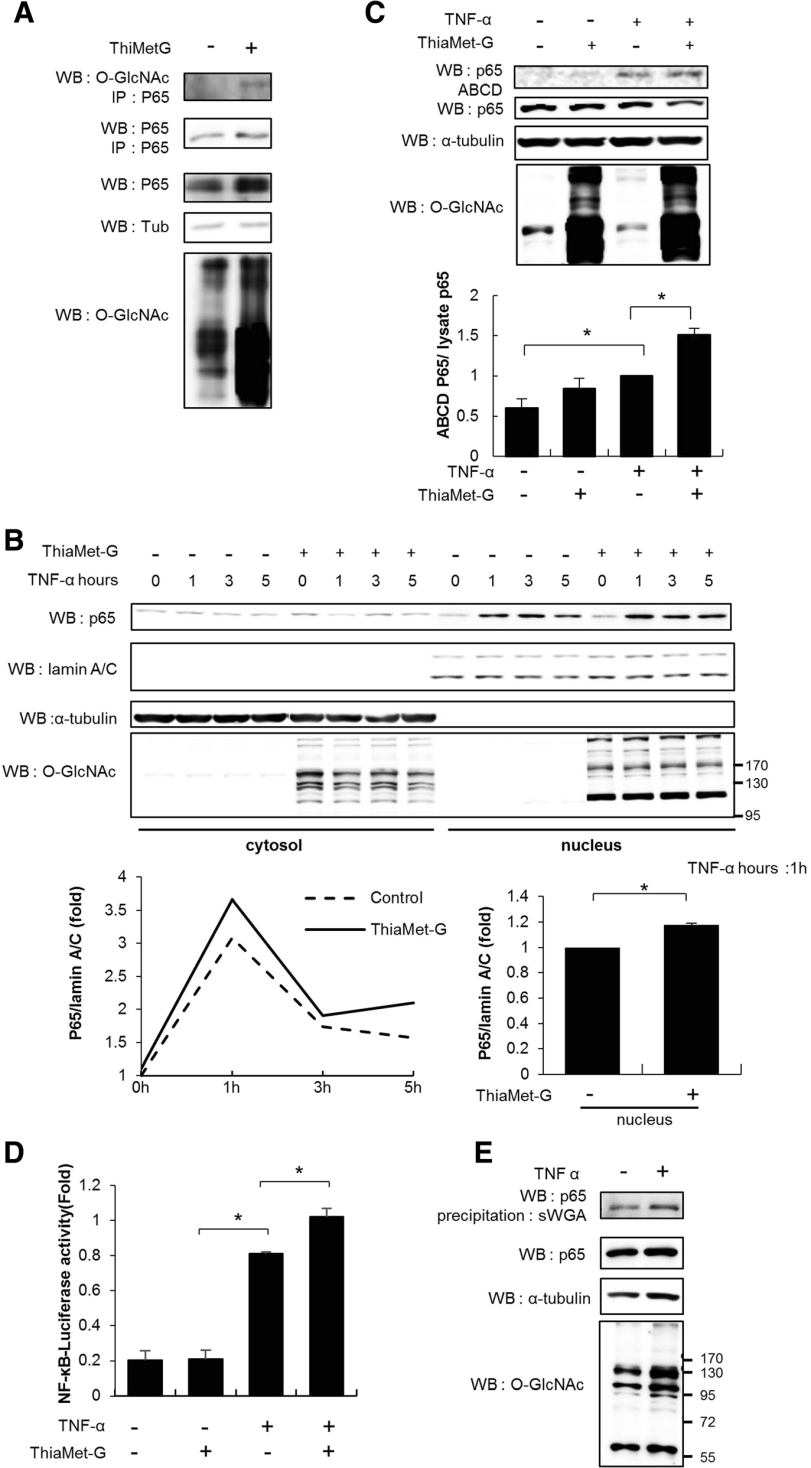
Nuclear localization, DNA binding affinity and transcriptional activity of O-linked N-acetylglucosamine glycosylated (*O-GlcNAcylated*) p65. **a** We confirmed that O-GlcNAcylation of p65 was enhanced by ThiaMet-G in MH7A cells using western blotting immunoprecipitation with both anti-p65 and anti-O-GlcNAc. **b** Western blotting (WB) was performed using anti-p65 and anti-O-GlcNAc and succinylated wheat germ agglutinin (sWGA)-affinity purification were performed for precipitating the O*-*GlcNAcylated proteins. ThiaMet-G significantly increased the nuclear translocation of p65 in TNF-α-simulated MH7A cells within 1 h. **c** and **d** Avidin-biotin complex DNA (*ABCD*) lysis buffer was used for MH7A cells lysis to measure DNA binding affinity. Lysates were incubated with biotinylated nuclear factor-kappa B (NF-κB) promoter site DNA. NF-κB transcriptional activity was measured in MH7A cells transfected with NF-κB-dependent luciferase reporter gene construct 4xkB- Luc. TNF-α-simulated MH7A cells exhibited significant increases in both the DNA binding affinity and transcriptional activity of p65, compared to controls and these effects were further enhanced following treatment with ThiaMet-G. **e** TNF-α alone augmented the level of O-GlcNAcylated p65 in fibroblast-like synoviocyte lysates. (*P <0.05)

We confirmed that O-GlcNAcylation of p65 was enhanced by ThiaMet-G in MH7A cells using western blotting immunoprecipitation with both anti-p65 and anti-O-GlcNAc (Fig. 2a). ThiaMet-G significantly increased the nuclear translocation of p65 in TNF-α-simulated MH7A cells within 1 h (Fig. 2b). TNF-α-simulated MH7A cells exhibited significant increases in both the DNA binding affinity and transcriptional activity of p65, compared to controls and these effects were further enhanced following treatment with ThiaMet-G (Fig. 2c and d). Moreover, TNF-α alone augmented the level of O-GlcNAcylated p65 in FLS lysates (Fig. 2e).

### The effect of hyper-O-GlcNAcylation on the severity of arthritis in mice with CIA

**Fig. 3:**
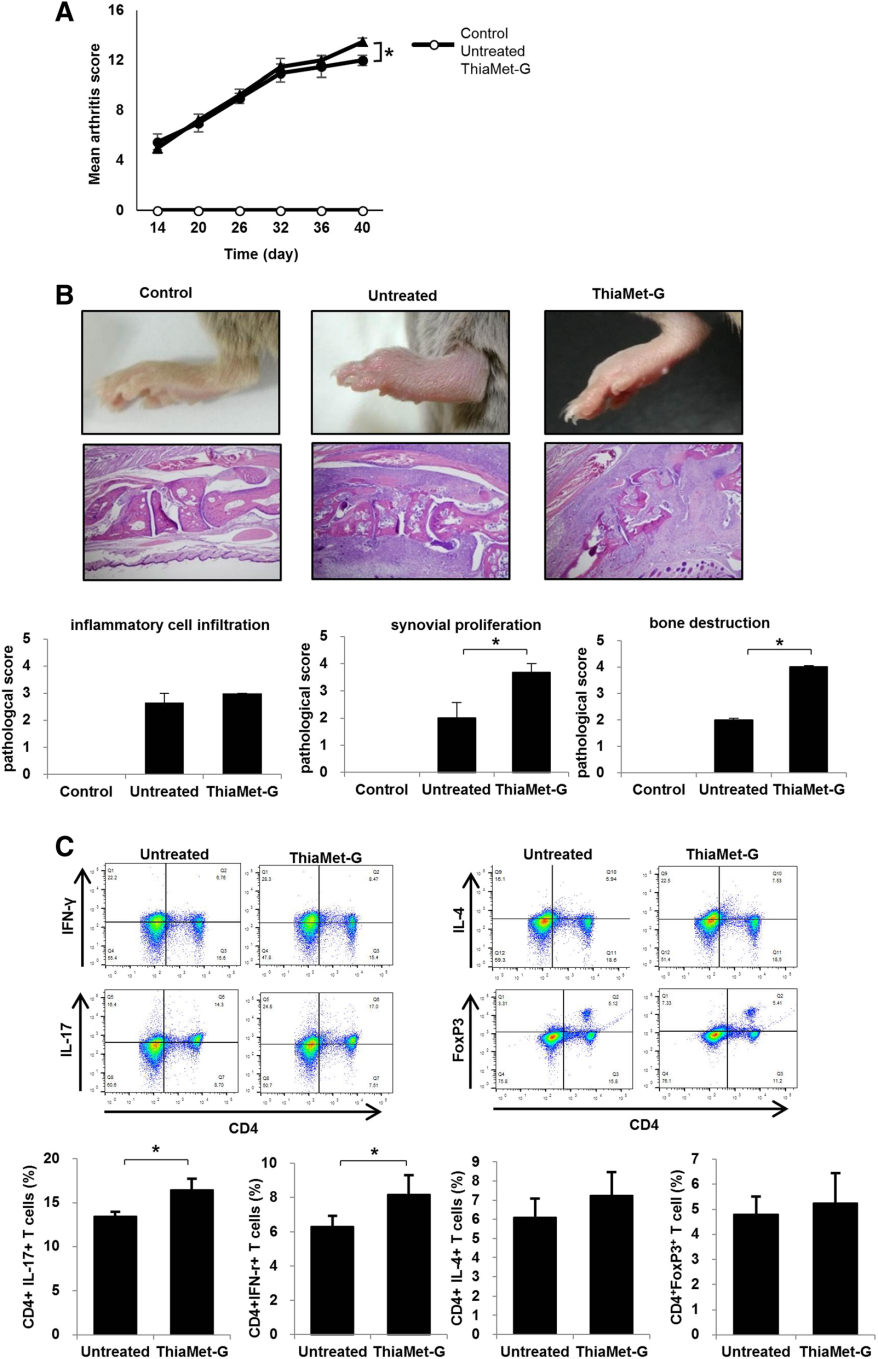
An animal model of rheumatoid arthritis (RA). Two weeks after primary immunization, mice with collagen-induced arthritis (CIA) were treated with 20 mg ThiaMet-G via intraperitoneal injection every other day for 20 days. Mice were then sacrificed and immune cells isolated from the spleen; T cells were then isolated and analyzed using fluorescence-activated cell sorting. **a** ThiaMet-G significantly aggravated the severity of arthritis in treated compared to untreated mice with CIA. **b** Histopathological evaluation of joint sections revealed that synovial proliferation and bone destruction, but not inflammatory cell infiltration, were more severe in mice with CIA treated with ThiaMet-G than in untreated mice (original magnification × 100). **c** ThiaMet-G significantly increased the numbers of CD4^+^ IFN-γ^+^ T cells (8.5 % vs 6.8 %) and CD4^+^ IL-17^+^ T cells (17.0 % vs 14.3 %), but not CD4^+^ IL-4^+^ T cells or CD4^+^ FoxP3^+^ T cells, in treated mice with CIA compared to untreated controls, respectively (*P <0.05)

ThiaMet-G significantly aggravated the severity of arthritis in CIA mice, compared to untreated mice with CIA (Fig. 3a). Histopathological evaluation of joint sections revealed that synovial proliferation and bone destruction, but not inflammatory cell infiltration, were more severe in ThiaMet-G-treated mice than untreated mice with CIA (Fig. 3b). On western blotting with anti-O-GlcNAc, ThiaMet-G remarkably enhanced the level of cellular O-GlcNAcylation in splenocytes of mice with CIA (Additional file 3). ThiaMet-G significantly increased the numbers of CD4^+^ IFN-γ^+^ T cells (8.5 % vs 6.8 %) and CD4^+^ IL-17^+^ T cells (17.0 % vs 14.3 %), but not CD4^+^ IL-4^+^ T cells or CD4^+^ FoxP3^+^ T cells, in treated compared to untreated mice with CIA, respectively (Fig. 3c). Our data suggested that hyper-O-GlcNAcylation by ThiaMet-G might drive T cell populations into T helper (Th)1 and Th17 cells, which play a pivotal role in the pathogenesis of RA.

## Discussion

We showed that hyper-O-GlcNAcylation accelerated the inflammatory responses of synoviocytes in vitro and CIA mice in vivo. Hyper-O-GlcNAcylation increased both cell proliferation and the expression levels of pro-inflammatory cytokines associated with RA in TNF-α-stimulated FLS. In contrast, hypo-O-GlcNAcylation in response to OGA overexpression significantly attenuated inflammation in TNF-α-stimulated FLS. Furthermore, we found that O-GlcNAcylation of p65 enhanced inflammatory processes in TNF-α-stimulated FLS via increased nuclear translocation of p65, DNA binding of the protein, and enhanced transcriptional activity, increasing the expression levels of downstream genes. Interestingly, TNF-α per se increased O-GlcNAcylation of p65 in the absence of OGA inhibitors, which may suggest that autocrine stimulation perpetuates inflammation in some patients.

In an animal model of RA, hyper-O-GlcNAcylation upon ThiaMet-G treatment significantly aggravated the severity of arthritis. In addition, hyper-O-GlcNAcylation increased the proportions of both Th1 and Th17 cells in mice with CIA, compared to untreated mice with CIA. Considering the therapeutic efficacy of p65 inhibitors in mice with CIA, and the ability of p65 to induce rearrangements in pro-inflammatory T cells [19, 20], data suggested that O-GlcNAcylation of p65 may exacerbate arthritis severity in part via driving T cell populations into Th1 and Th17 cells, which play a pivotal role in the pathogenesis of RA.

O-GlcNAc modification is often suggested to serve as a nutrient sensor, being more common in hyperglycemic conditions (diabetes mellitus and metabolic syndrome). Here, we focused on the role of O-GlcNAcylation in RA, although any direct correlation between hyperglycemia and RA disease activity has yet to be elucidated. We studied local joint hypoxia rather than systemic glycemic conditions, because uncontrolled synovial hyperplasia triggered by various pro-inflammatory cytokines may induce local hypoxia-like cancers, which in turn may trigger hyper-O-GlcNAcylation [21].

With these results, we might carefully suggest a hypothesis on the association between hyper-O-GlcNAcylation or O-GlcNAcylation of p65 and the aggravated severity of arthritis as follows: (i) upon initiation of RA, hyperplasia of the synovium may create localized hypoxia; (ii) in turn there might be a status of hyper-O-GlcNAcylation including p65; (iii) these effects are further exacerbated by TNF-α per se, increasing O-GlcNAcylation of p65 further; (iv) O-GlcNAcylation of p65 enhances nuclear translocation and transcriptional activation, increasing the expression levels of pro-inflammatory molecules; (v) these pro-inflammatory molecules augment the synovial inflammation, thereby enhancing T cell-mediated inflammation, resulting in RA aggravation quite apart from the effects of O-GlcNAcylation of p65 (Additional file 4).

We had several limitations in our pilot study. We did not explore the effects of O-GlcNAcylation on other kinases and transcriptional factors involved in RA pathogenesis and we did not use the minimal dose of ThiaMet-G for cultured cells. We used the relatively high concentration of ThiaMet-G to investigate the effect of ThiaMet-G treatment in TNF-α-stimulated synovial cells. A future study including such work will improve our understanding of O-GlcNAc-mediated inflammation, but data presented here provide valuable evidence for a role of p65 O-GlcNAcylation of p65 in RA.

## Conclusion

We herein showed that O-GlcNAcylation of p65 increased the effects of TNF-α-mediated inflammation both in vitro (in synovial cells) and in vivo (in mice with CIA). We believe that our study may serve as a starting point for future investigations into the role played by O-GlcNAcylation in RA.

## Additional files

Additional file 1:
**Primers for** **RT-PCR.** A full list of primers for inflammatory molecules used in RT-PCR. (DOC 30 kb) Additional file 2:
**MTT assay.** TNF-α (10 μg/mL) significantly increased proliferation of fibroblast-like synoviocytes (FLS), compared to controls and proliferation was further enhanced following treatment with by NButGT (50 μM, for 24 h). (JPEG 79 kb) Additional file 3:
**ThiaMet-G-treated splenocytes.** ThiaMet-G remarkably enhanced the level of cellular O-GlcNAcylation level in splenocytes of mice with collagen-induced arthritis on western blotting with anti-O-GlcNAc. (JPEG 102 kb) Additional file 4:
**Hypothesis of the link between hyper-** **O-GlcNAcylation** **and the aggravation in arthritis severity.** (i) Upon initiation of rheumatoid arthritis, hyperplasia of the synovium may create localized hypoxia; (ii) in turn there might be a status of hyper-O-GlcNAcylation including p65; (iii) these effects are further exacerbated by TNF-α per se, increasing O-GlcNAcylation of p65 further; (iv) O-GlcNAcylation of p65 enhances nuclear translocation and transcriptional activation, increasing the expression levels of pro-inflammatory molecules; (v) these pro-inflammatory molecules augment the synovial inflammation, thereby enhancing T cell-mediated inflammation, resulting in aggravation of rheumatoid arthritis quite apart from the effects of O-GlcNAcylation of p65. (JPEG 276 kb)

## Abbreviations

ABCD: avidin-biotin complex DNA
CCL5: chemokine ligand 5
CIA: collagen-induced arthritis
FACS: fluorescence-activated cell sorting
FLS: fibroblast-like synoviocytes
IFN: interferon
IL: interleukin
MMP-1: matrix metalloproteinase-1
NF-κB: nuclear factor-kappa B
OGA: O-linked N-acetylglucosaminidase
O-GlcNAc: O-linked N-acetylglucosamine
O-GlcNAcylation: O-linked N-acetylglucosamine glycosylation
OGT: O-linked N-acetylglucosamine transferase
PBS: phosphate-buffered saline
RA: rheumatoid arthritis
RT-PCR: reverse transcription PCR
sh-Luc: sh-luciferase
shRNA: small hairpin RNA
sWGA: succinylated wheat germ agglutinin
TNF: tumor necrosis factor
