# Supplementary material for: O-linked N-acetylglucosamine glycosylation of p65 aggravated the inflammation in both fibroblast-like synoviocytes stimulated by tumor necrosis factor-α and mice with collagen induced arthritis
Source: Arthritis Res Ther. 2015 Sep 14;17(1):248. doi: 10.1186/s13075-015-0762-7 (PMC4570085; doi:10.1186/s13075-015-0762-7)
Supplement: Additional file 1: — Primers for RT-PCR. A full list of primers for inflammatory molecules used in RT-PCR. (DOC 30 kb) [file 13075_2015_762_MOESM1_ESM.doc]

**Additional file 1. Primers for RT-PCR**

| MMP-1 | forward | 5′-CCTAGCTACACCTTCAGTGG |
| --- | --- | --- |
|  | reverse | 5′-GCCCAGTACTTATTCCCTTT |
| CCL5 | forward | 5′-CCTCATTGCTACTGCCCTCT |
|  | reverse | 5′-GGTGTGGTGTCCGAGGAATA |
| IL-6 | forward | 5′-CCCCCAGGAGAAGATTCCAA |
|  | reverse | 5′-GCTGCTTTCACACATGTTACTCTTG |
| IL-8 | forward | 5′-ACTTTCAGAGACAGCAGAGC |
|  | reverse | 5′-GTGGTCCACTCTCAATCACT |
