# Supplementary figures and images for: O-linked N-acetylglucosamine glycosylation of p65 aggravated the inflammation in both fibroblast-like synoviocytes stimulated by tumor necrosis factor-α and mice with collagen induced arthritis
Source: Arthritis Res Ther. 2015 Sep 14;17(1):248. doi: 10.1186/s13075-015-0762-7 (PMC4570085; doi:10.1186/s13075-015-0762-7)

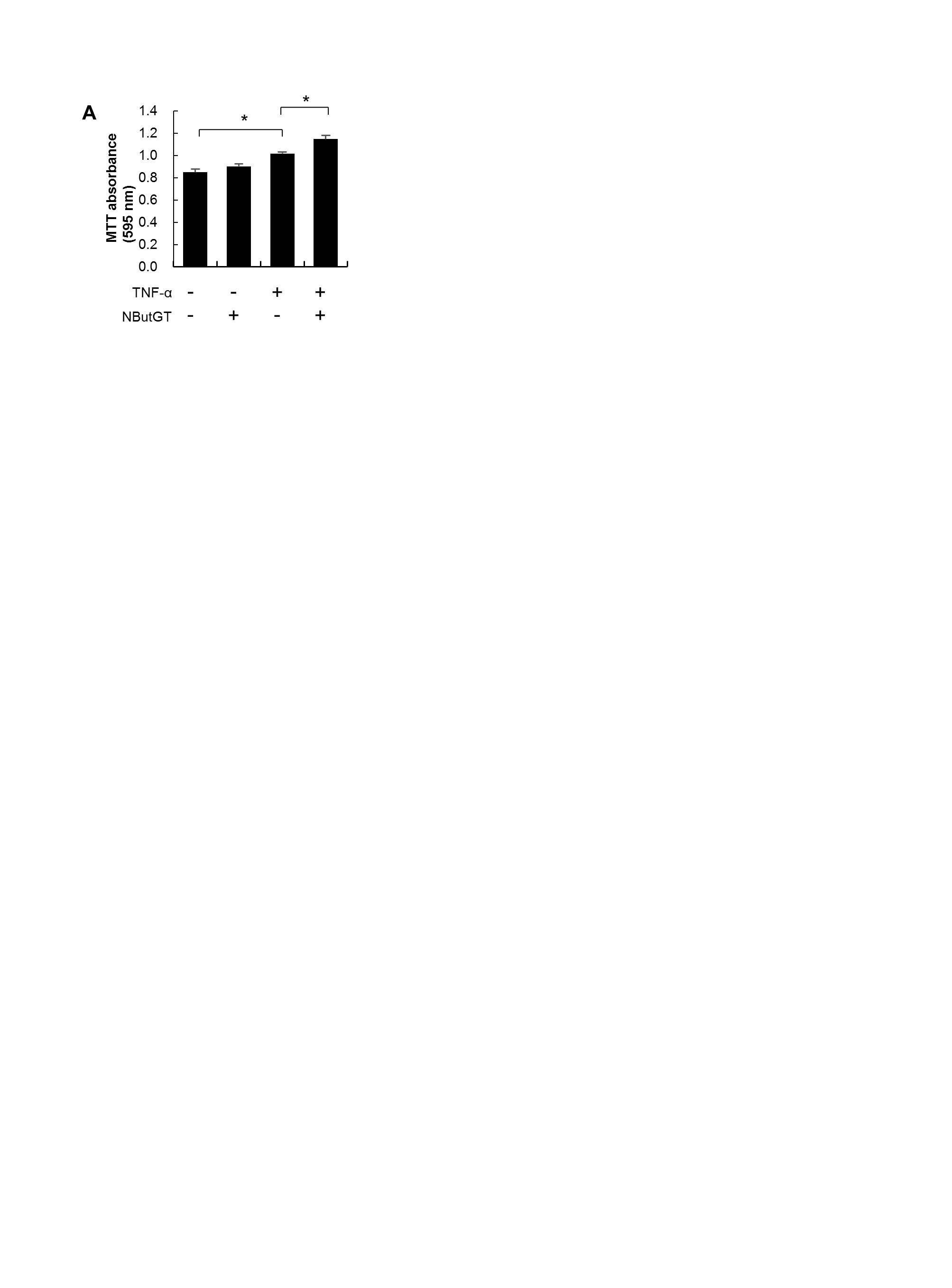

Supplement: Additional file 2: — MTT assay. TNF-α (10 μg/mL) significantly increased proliferation of fibroblast-like synoviocytes (FLS), compared to controls and proliferation was further enhanced following treatment with by NButGT (50 μM, for 24 h). (JPEG 79 kb) [file 13075_2015_762_MOESM2_ESM.jpeg]

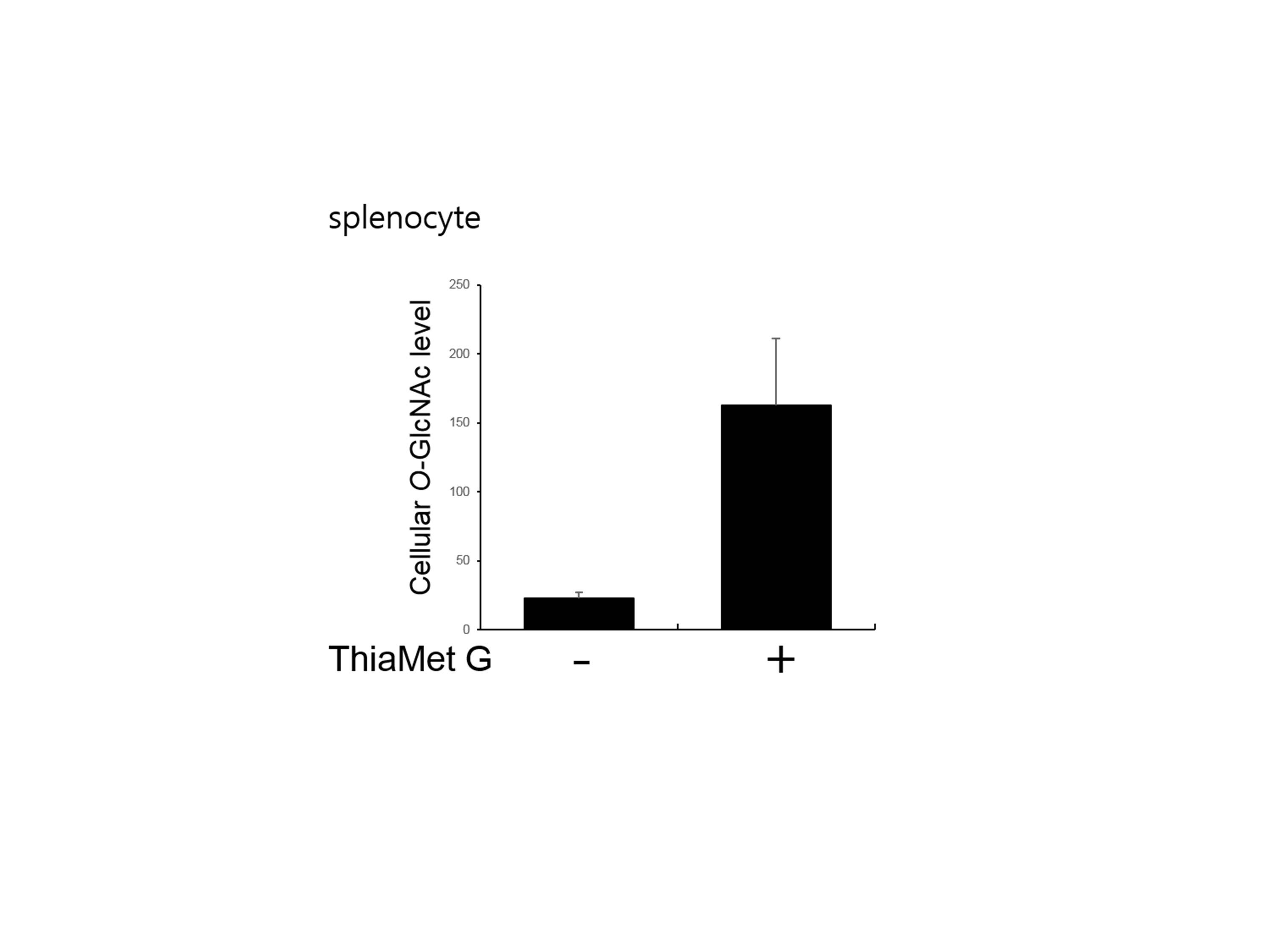

Supplement: Additional file 3: — ThiaMet-G-treated splenocytes. ThiaMet-G remarkably enhanced the level of cellular O-GlcNAcylation level in splenocytes of mice with collagen-induced arthritis on western blotting with anti-O-GlcNAc. (JPEG 102 kb) [file 13075_2015_762_MOESM3_ESM.jpeg]

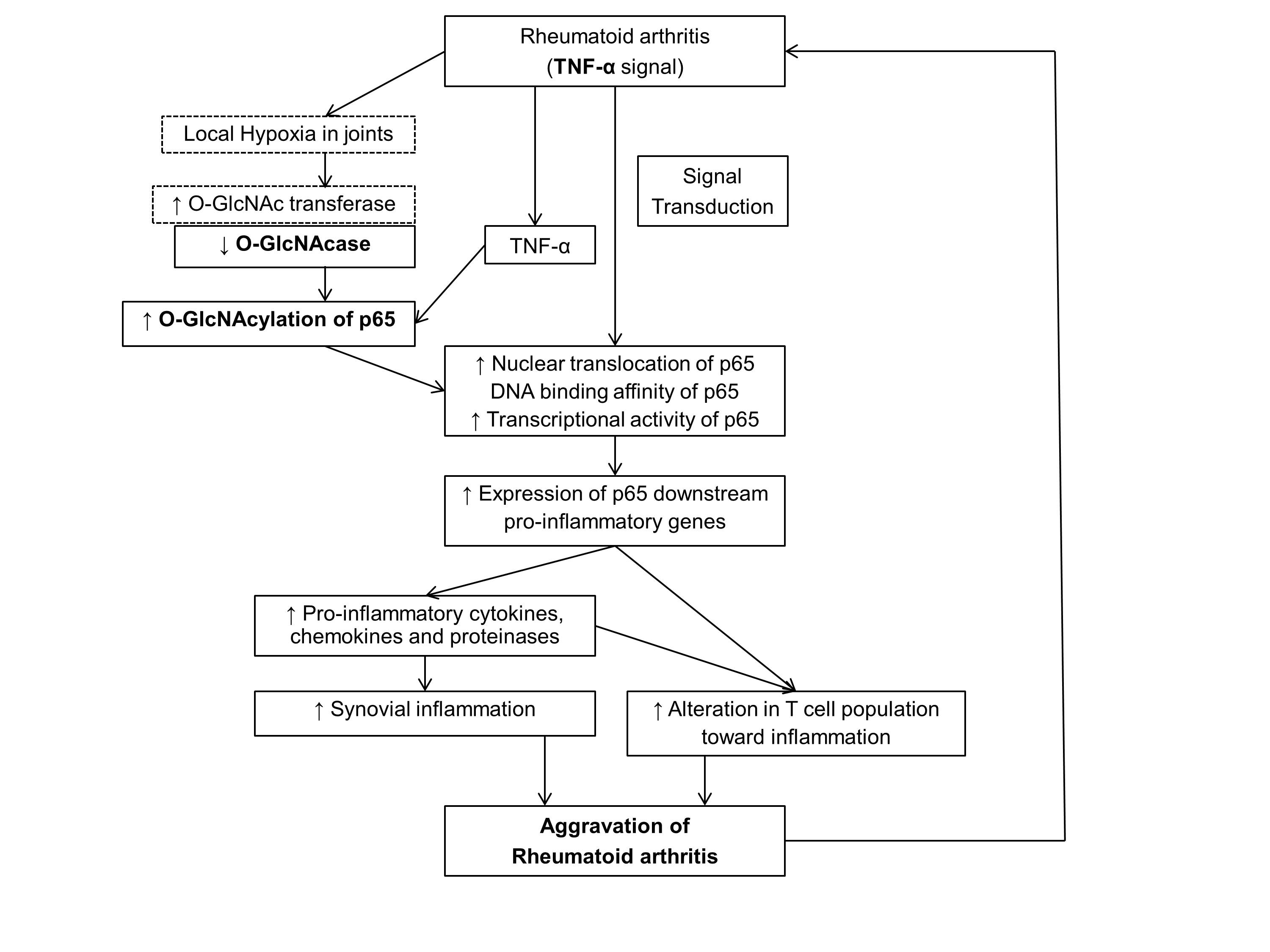

Supplement: Additional file 4: — Hypothesis of the link between hyper- O-GlcNAcylation and the aggravation in arthritis severity. (i) Upon initiation of rheumatoid arthritis, hyperplasia of the synovium may create localized hypoxia; (ii) in turn there might be a status of hyper-O-GlcNAcylation including p65; (iii) these effects are further exacerbated by TNF-α per se, increasing O-GlcNAcylation of p65 further; (iv) O-GlcNAcylation of p65 enhances nuclear translocation and transcriptional activation, increasing the expression levels of pro-inflammatory molecules; (v) these pro-inflammatory molecules augment the synovial inflammation, thereby enhancing T cell-mediated inflammation, resulting in aggravation of rheumatoid arthritis quite apart from the effects of O-GlcNAcylation of p65. (JPEG 276 kb) [file 13075_2015_762_MOESM4_ESM.jpeg]
